# Antipsychotics in Alzheimer’s disease: A critical analysis

**DOI:** 10.1590/S1980-57642011DN05010007

**Published:** 2011

**Authors:** Eduardo Marques da Silva, Rafaela de Castro Oliveira Pereira Braga, Thiago Junqueira Avelino-Silva, Luiz Antonio Gil Junior

**Affiliations:** Department of Geriatrics, Clinical Hospital of The University of São Paulo, São Paulo SP, Brazil.

**Keywords:** antipsychotics, dementia, side effects, Alzheimer, neuropsychiatric symptoms

## Abstract

The estimated worldwide prevalence of dementia among adults older than 60 years of age was 3.9% in 2005. About 90% of demented patients will develop neuropsychiatric symptoms (NS) such as delirium, delusion, aggressiveness and agitation. The treatment of NS involves non-pharmacologic strategies (with varying degrees of success according to the scientific literature) and pharmacologic treatment (PT). The present review of literature examined the current role of AP in the management of NS in dementia.

**Methods:**

A thematic review of medical literature was carried out.

**Results:**

313 articles were found, 39 of which were selected for critical analysis. Until 2005, the best evidence for PT had supported the use of selective serotonin re-uptake inhibitors (SSRIs), anticholinesterases, memantine and antipsychotics (AP). In 2005, the U.S. Food and Drug Administration (FDA) disapproved the use of atypical APs to treat neuropsychiatric symptoms in individuals with dementia (the same occurred with the typical APs in 2008). After this, at least two important randomized placebo-controlled multicenter trials were published examining the effectiveness of atypical APs in Alzheimer’s disease (CATIE-AD) and the effects of interrupting AP treatment (DART-AD).

**Conclusions:**

Based on the current evidence available, APs still have a place in treatment of the more serious psychotic symptoms, after the failure of non-pharmacological treatment and of an initial approach with selective inhibitors of serotonin uptake, anticholinesterases and memantine.

## Abstract (pt)

A prevalência mundial de demencia entre idosos foi de 3,9% em 2005. Cerca de 90% dos dementados apresentarão sintomas como delirium, alucinações, agressividade e agitação. O tratamento dos sintomas não cognitivos envolve estratégias não farmacológicas – com sucesso variável de acordo com a literatura – e tratamento medicamentoso. A presente revisão de literatura discute o atual papel dos antipsicóticos nos sintomas neurocomportamentais de demência.

**Métodos:**

Conduzimos uma revisão temática nas principais bases de dados da literatura.

**Resultados:**

313 artigos sobre o tema foram encontrados e 39 selecionaos para compor uma análise crítica. Até 2005, as melhores evidências para tratamento medicamentoso indicavam o uso de inibidores da acetilcolinesterase memantina e antipsicóticos. Em 2005 o FDA desautorizou o uso de antipsicóticos atípicos em dementados (em 2008 fez o mesmo com os típicos). Após isto, 2 ensaios clínicos randomizados placebo-controlados foram concluídos avaliando a eficácia dos antipsicóticos atípicos em portadores de doença de Alzheimer (CATIE-AD) e os efeitos da interrupção destes (DART-AD).

**Conclusões:**

Considerando as evidências atuais, os antipsicóticos ainda têm importância no manejo dos sintomas psicóticos mais sérios, após falha de tratamento não farmacológico e de uma abordagem inicial com inibidores de receptação de serotonina, anticolinesterásicos e memantina.

The estimated worldwide prevalence of dementia among adults older than 60 years old was 3.9% in 2005.^1^ According to 2008 data, the prevalence in São Paulo (Brazil) was 12.9%, and Alzheimer’s disease (AD) was responsible for 59.8% of all dementias.^2^ AD is a lethal progressive neurodegenerative disorder, and is characterized by cognitive decline, loss of capacity to execute daily activities, and a variety of behavioral symptoms.^3^

About 90% of demented patients will develop neuropsychiatric symptoms (NPS) such as delirium, delusion, aggressiveness and agitation (78.33% of Brazilian elders).^45^ These NPS eventually precipitate institutionalization,^6^ functional decline,^7^ and contribute to caregiver stress and depression.^8,9^ The treatment of AD neuropsychiatric symptoms has been the target of multiple lines of research in recent years. Non-pharmacologic strategies have been shown to work with variable success^10,11^ and include the use of music,^12-14^ aromatherapy,^15^ pet therapy and family members videos.^16-18^ Pharmacologic interventions include agents such as benzodiazepines, anticholinesterases, memantine, antidepressants, mood stabilizers, anticonvulsants and antipsychotics (AP). In addition, it is known that the prescription of drugs for NPS in dementia is influenced by a variety of factors, including caregiver stress.^19^ The objective of this article was to discuss the latest evidence examining the management of neuropsychiatric symptoms in dementia with an emphasis on neuroleptics.

## Methods

In preparing for this review, a search of the literature from 1999 to September 2009 of several electronic bases (Medline, PubMed, ScieLO, LILACS, and the Cochrane Library) was carried out. Keywords used individually and in various combinations included: antipsychotics, dementia, Alzheimer’s disease and neuropsychiatric symptoms. The filters used were double-blind, placebo-controlled, randomized trials (RCT), meta-analysis, case series and review articles. The references generated were checked and analysed for the qualitative relevance on the basis of their title and abstract, while other references drawn from the papers identified were followed up.

## Results

The search method retrieved 313 articles. Of these, 79 were RCTs, 59 non-randomized trials, 9 meta-analyses, 60 reviews and 106 case series. A total of 39 articles were selected based on their qualitative relevance.

## Discussion

In 2005, a significant review of the pharmacological treatment of NPS in dementia^20^ was published. The best evidence from this report had supported the use of selective serotonin re-uptake inhibitors (SSRIs), anticholinesterases, memantine and APs. SSRIs were effective in the control of anxious and depressive symptoms, with additional evidence needed to confirm the advantage of citalopram for the treatment of psychotic symptoms.^21,22^ Anticholinesterases and memantine showed limited, although significant, effect in more highly symptomatic patients.^23^ Recently, the results of a placebo-controlled randomized trial revealed no difference between 10mg of donepezil and placebo on the Cohen–Mansfield Agitation Inventory (CMAI).^24^ Well-designed studies on anticonvulsants are scarce, and no randomized trials with good follow up are available, while the overriding impression is that adverse effects (such as sedation and drowsiness) often outweigh benefits.^25-29^

APs are among the most used medications for the control of symptoms such as agitation and aggressiveness associated with dementia.^30^ Up to the end of 2009, the effectiveness of typical antipsychotic agents had been tested in 8 clinical placebo-controlled trials,^30,31^ while atypical agents were tested in 20 trials.^32-38^ In addition, a Finnish study verified that the prescription of these agents in nonagenarians has been due mainly to inappropriate social behaviors, recurrent anxious complaints and repetitive physical movements.^39^

The growing interest in atypical APs (i.e., risperidone, clozapine, olanzapine, quetiapine, ziprasidone, sulpiride and aripiprazol) has arisen due to the frequent number of adverse events observed with the use of typical agents (i.e., chlorpromazine, levomepromazine, haloperidol and thioridazine) described as causing predominantly extrapyramidal symptoms. Other adverse effects of typical antipsychotics use include tardive dyskinesia, neuroleptic malignant syndrome, weight gain, dystonia, hyperglycemia, hypotension, dyslipidemia, QT prolongation, akathisia, hyponatremia, lethargy, anticholinergic effects and drowsiness. Nevertheless, even though it is known that the incidence of parkinsonism is lower with atypical agents, a retrospective study involving more than 25,000 individuals receiving high doses of atypical agents verified a surprisingly high incidence of parkinsonism.^40^ Moreover, femur fracture was correlated with the use of AP in a case-control study in 2007,^41^ indicating caution in patients with an increased risk for falls, encompassing almost all demented patients.

Meta-analysis of studies evaluating adverse effects of AP medications verified an increased incidence of stroke and transient ischemic attack (TIA) with the use of risperidone and olanzapine.^42-45^ Nevertheless, a case-controlled study with almost 4,000 individuals^46^ and another retrospective study in more than 11,000 people^47^ did not confirm this association. These conflicting results raise the question: should prescriptions of AP be limited.^48^ In 2005, a meta-analysis of 15 studies^49^ showed increased mortality with AP administration but this rise did not achieve statistical significance when each study was considered separately. In 2005, the U.S. Food and Drug Administration (FDA) disapproved the use of atypical AP to treat neuropsychiatric symptoms in individuals with dementia. This warning was based on the analysis of 17 placebo-controlled clinical assays, which involved 5,377 elderly individuals (with dementia and behavioral symptoms) and showed an increase in mortality rates of 1.6 to 1.7.^50^ In June 2008, the FDA extended this recommendation to typical antipsychotic agents^51^ based on two retrospective studies involving a total of 64,000 patients.^52,53^ The increase in mortality among these subjects was attributed to cardiovascular events and infections, which mostly occurred in the respiratory system.^54^

The recommendations prior to 2006 had indicated the use of APs only after a lack of response to treatment with non-pharmacological measures, anticholinesterases and memantine.^20,55^ These recommendations had emphasized that randomized prospective studies with larger samples and over a longer period of time would be necessary to improve evidence supporting the prescription of APs. In 2006 and 2008, two important randomized placebo-controlled multicenter trials were published examining the effectiveness of atypical APs in Alzheimer’s disease and the effects of interrupting AP treatment. These studies were denominated the CATIE-AD (Clinical Antipsychotic Trials of Intervention Effectiveness Alzheimer’s Disease)^38^ and the DART-AD (Dementia antipsychotic withdrawal Trial-Alzheimer’s Disease).^35,36^

### CATIE-AD

The CATIE-AD was a clinical study conducted in patients from 45 centers. Older individuals diagnosed with AD and neuropsychiatric symptoms were randomized into groups receiving olanzapine, quetiapine, risperidone or placebo. Patients with other forms of dementia were excluded, as were those who had been treated with anticholinesterasics or antidepressants, or had been institutionalized. The primary endpoint was the time until discontinuation of the medication for any reason (phase 1). The secondary endpoints were: improvement on the Clinical Global Impression of Change (CGIC) scale and the time until discontinuation due to ineffectiveness or intolerance. All individuals that used at least one dose were analyzed according to intention to treat. In the second phase (phase 2), participants whose treatment had been interrupted in phase 1 were randomized into another group (except for the previous group) or received citalopram. Of the 421 randomized elderly patients, 63% had discontinued the medication by the 12^th^ week and 82% by the 36th week of treatment. There was no significant difference in the primary outcome between the medicated and placebo groups.

The results of this study demonstrated that olanzapine and risperidone were significantly more effective than quetiapine and placebo when interruption of treatment due to a lack of efficacy was analyzed. The placebo group was superior to the three drugs on the analysis of treatment interruption due to intolerability. With regard to the CGIC, 32% of the patients in the olanzapine group, 29% of the risperidone group, 26% of the quetiapine group and 21% of the placebo group improved (although the differences among these groups were not statistically significant). Extrapyramidal adverse effects were more marked in the olanzapine and risperidone groups and frequently led to interruption of treatment. A recent publication^51^ based on the original CATIE-AD study showed that olanzapine was correlated with a decrease in high-density lipoproteins (HDL). Sedation and weight gain were less common in the placebo group. In addition, the Mini Mental Status Examination (MMSE) score did not worsen with the use of antipsychotics. However, a limitation of this study was that the participants’ doctors frequently did not adjust the medication dose when the goals of treatment had not been achieved, which goes against good clinical practice. Also, the previous use of APs was not considered in the experimental protocol.

It was not the objective of the cited publication to comment on the results of the phase 2 study.^38^ A second publication^57^ interpreted the data obtained from the same 421 patients and evaluated the following measures: Alzheimer’s Disease Cooperative Study (Clinical Global Impression of Change (ADCS-CGIS), Brief Psychiatric Rating Scale (BPRS), Scale of Cornell for Depression in Insanity, Alzheimer’s Disease Cooperative Study - Activities of Daily Living Scale (ADCS-ADLS), Caregiver Activity Survey, Dependence Scale and Alzheimer’s Disease Related Quality of Life. These parameters were checked at weeks 0, 2, 4, 8, 12, 24 and 36. However, these measurements were limited to phase 1 of the study. Hence, there were two analyses, namely, one during the last interview that was conducted before the patient discontinued the medication, and another at the 12^th^ week.

The results of these two analyses were similar. A benefit was evident for the olanzapine and risperidone groups in NPI (patients improved by 7 and 11.6 points, respectively) and on the BPRS sub-item for hostile distrust. A benefit was also seen for the risperidone group on the ADCS-CGIS and BPRS sub-item for psychosis at the end of phase 1. By contrast, the olanzapine group exhibited a significantly poorer outcome on the ADCS-ADLS and BPRS sub-item for withdrawn depression factor. Comparing the other scales, no statistical difference was detected among the groups. Analysis of the characteristics of the studied groups showed that the group randomized for olanzapine presented with a lower NPI during the initial phases of the study. In the group analyzed at the 12^th^ week, 63% of the participants had discontinued the medication (due to intolerability or lack of efficacy).

### DART-AD

The DART-AD study^35^ was a clinical randomized placebo-controlled assay evaluating the discontinuation of treatment with APs. The inclusion criteria for this study were use of thioridazine, chlorpromazine, haloperidol, trifluorperazine or risperidone for at least 3 months; meeting the criteria of NINCDS/ADRDA as possible or probable AD with a MMSE greater than 6; and use of at least the equivalent of 10 mg of chlorpromazine or 0.5 mg of risperidone. The selected patients were randomized to continue the treatment or to switched to placebo. The protocol excluded individuals unable to complete the initial evaluation or whose participation in the study could have led to an increase in suffering according to the researchers’ opinion. The primary endpoint was worsening on the Severe Impairment Battery (SIB). Secondary endpoints included the Neuropsychiatric Inventory (NPI), the Functional Assessment Staging (FAS) test, and verbal fluency test. In the study, 220 participants were assessed and 165 were effectively randomized. Of the 165 patients, 102 started the protocol and only 77 remained until the end of the 6-month period in their originally allocated group.

The primary endpoint (SIB) at the 6^th^ and 12^th^ month did not differ between the studied groups. However, a difference of 8 points favoring the placebo group was observed at the 12^th^ month. The NPI was similar at the 6^th^ and 12^th^ month, but separation of these individuals according to their initial NPI (with a range of 14 points) led to a statistically significant difference, favoring the group that continued with AP, by more than 14 points. The FAS score was statistically different and favored the placebo group at the end of the 6-month evaluation period. The authors also suggested that analysis with additional exploratory sensitivities did not alter the published results, nor did the inclusion of patients with MMSE scores of less than 6. In spite of these caveats, the chance of type II error and the occurrence of treatment disruption were considered high.

About one year later, data from the same sample was published that analyzed up to 52 months of continuous evaluation with mortality as the primary endpoint.^36^ The statistical analysis considered intention to treat, which included those patients that had used at least a single medication dose or placebo, as well as individuals that did not get to begin the allocated treatment (excluding the subjects that had interrupted their treatment before 12 months). This analysis also included patients with a modified intention to treat (including all patients that used at least one medication dose or placebo). The results from this study showed a mortality rate increase in the group that continued with AP, particularly after the first year. The accumulated survival percentages for the AP and placebo groups were as follows: to the 24^th^ month, 46% versus 71%; to the 36^th^ month, 30% versus 59%; and to the 42^nd^ month, 26% versus 53%, respectively. The analysis for intention to treat or modified intention to treat presumed that the patients stayed in the originally allocated group for at least 12 months. Death Certificates for 78% of these patients were reviewed, and did not reveal an increased incidence of cerebrovascular disease in the group that continued receiving AP. However, once the study was accomplished, a telephone follow-up was conducted to assess mortality rates only after the first 12 months of treatment. Therefore, a limitation of this analysis was the lack of information regarding the subsequent antipsychotics’ prescription at the end of the 12-month period of the protocol for the placebo group (or the incidence of retreatment in the group that received AP for 12 months). Another limitation was the exclusion of individuals with an MMSE score of less than 6, whom had a greater risk of death. The authors suggest caution in interpreting these data, especially considering that few participants were analyzed at the later time points in the study.

Considering the current evidence available, APs still have a place in the treatment of the more serious psychotic symptoms associated with dementia, such as anger, aggression, and paranoid ideas, although they do not appear to improve functioning, care needs, or quality of life. After the failure of non-pharmacological treatment as an initial approach to solve these symptoms, and of selective inhibitors of serotonin uptake, anticonvulsants, anticholinesterases and memantine, the lack of safer alternatives enforces the use of antipsychotics for dementia neuropsychiatric symptoms. In addition, there is reasonable evidence favoring the use of atypical agents over typical ones, even though no one specific agent has been defined as the drug of choice based on the available literature. There is an urgent need for novel alternative therapeutics.
